# Dielectric Properties of Colossal-Dielectric-Constant Na_1/2_La_1/2_Cu_3_Ti_4_O_12_ Ceramics Prepared by Spark Plasma Sintering

**DOI:** 10.3390/molecules27030779

**Published:** 2022-01-25

**Authors:** Hicham Mahfoz Kotb, Mohamad Mahmoud Ahmad, Sajid Ali Ansari, Tarek S. Kayed, Adil Alshoaibi

**Affiliations:** 1 Department of Physics, College of Science, King Faisal University, Al-Hassa 31982, Saudi Arabia; mmohamad@kfu.edu.sa (M.M.A.); sansari@kfu.edu.sa (S.A.A.); adshoaibi@kfu.edu.sa (A.A.); 2 Department of Physics, Faculty of Science, Assiut University, Assiut 71516, Egypt; 3 Department of Physics, Faculty of Science, The New Valley University, El-Kharga 72511, Egypt; 4 Department of Basic Engineering Sciences, College of Engineering, Imam Abdulrahman Bin Faisal University, Dammam 34221, Saudi Arabia; tkayed@iau.edu.sa

**Keywords:** colossal dielectric constant, spark plasma sintering (SPS), NLCTO, CCTO

## Abstract

In the current study, we report on the dielectric behavior of colossal-dielectric-constant Na_1/2_La_1/2_Cu_3_Ti_4_O_12_ (NLCTO) ceramics prepared by mechanochemical synthesis and spark plasma sintering (SPS) at 850 °C, 900 °C, and 925 °C for 10 min. X-ray powder diffraction analysis showed that all the ceramics have a cubic phase. Scanning electron microscope observations revealed an increase in the average grain size from 175 to 300 nm with an increase in the sintering temperature. SPS NLCTO ceramics showed a room-temperature colossal dielectric constant (>10^3^) and a comparatively high dielectric loss (>0.1) over most of the studied frequency range (1 Hz–40 MHz). Two relaxation peaks were observed in the spectra of the electrical modulus and attributed to the response of grain and grain boundary. According to the Nyquist plots of complex impedance, the SPS NLCTO ceramics have semiconductor grains surrounded by electrically resistive grain boundaries. The colossal dielectric constant of SPS NLCTO ceramics was attributed to the internal barrier layer capacitance (IBLC) effect. The high dielectric loss is thought to be due to the low resistivity of the grain boundary of SPS NLCTO.

## 1. Introduction

Colossal-permittivity (CP) materials (ε′ > 10^3^) have potential uses in several technological applications, such as multilayer ceramic capacitors and memory devices [1]. Intensive studies on CP materials during the last two decades have given rise to several material families with CP properties. The titanate families such as CaCu_3_Ti_4_O_12_ (CCTO) [2,3,4], doped TiO_2_, and their derived ceramics [5,6,7,8,9,10,11,12] are still attracting the attention of researchers. Generally, the structural, electrical, and dielectric properties of CP materials are found to be sensitive to the powder synthesis, calcination, and sintering methods and conditions. Conventional solid-state-reaction (SSR) method is still by far the most used technique for the preparation of CP ceramics [5,13]. Nevertheless, the SSR method has the drawbacks of being a long, high-temperature process with less control on the resulting grain size of the ceramic. Recently, promising results have been reported after successful combinations of innovated powder synthesis processes, e.g., sol–gel [6]; autocombustion [14]; and alternating sintering methods, i.e., microwave [15] and spark plasma sintering (SPS) [3,8,16,17]. Compared to the standard SSR process, other methods aim to simplify the fabrication process and to reduce the maximum temperature and the time of the process without scarifying the dielectric properties of the ceramic. SPS is a comparatively new sintering technique that has the advantages of being fast due to the rapid heating rates and short dwelling times, which may lead to good control on the grain size of the resulting ceramics [17]. In the SPS process, the as-prepared powder is usually loaded in a graphite die-punch set and pressurized mechanically using a controllable isostatic pressure on the graphite punch. Afterward, a pulsed current is passed through the punch-die setup in order to heat the sample. The whole SPS process takes place under vacuum in most cases. SPS was deployed successfully to prepare ceramic materials such as CaCu_3_Ti_4_O_12_ [17], BaTiO_3_ [18], Ba(Fe_1/2_Nb_1/2_)O_3_ [19], and SrZrO_3_ [20] at comparatively low temperatures. In the present work, we report on the dielectric properties of the Na_1/2_La_1/2_Cu_3_Ti_4_O_12_ (NLCTO) ceramics. NLCTO is a promising CP material that exhibited ε′~6.1–8.7 × 10^3^ when prepared by the SSR process that included two calcinations steps, at 950 °C for 15 h and at 1000 °C for 10 h, and conventional sintering at 1080–1090 °C for 5 h. In another study, the powder of NLCTO was first prepared by sol–gel and then conventionally sintered at 1060–1100 °C for 5–15 h. The resulting ceramics exhibited ε′~1.1–1.8 × 10^4^ [21]. Previously, we reported on the colossal dielectric properties of NLCTO ceramics prepared by the mechanomechanical synthesis of the powder and reactive sintering inside a conventional tubular furnace at 1025–1100 °C for 10 h [22]. The obtained ceramics showed ε′~3.8–9.6 × 10^3^ at 1.1 KHz. In the present study, we investigate the dielectric properties of NLCTO ceramics where the powder was first synthesized by mechanochemical milling and then the SPS method was used to obtain the dense, pure-phase ceramics. X-ray diffraction (XRD) and a field-emission scanning electron microscope (FE-SEM) were used to investigate the phase purity and microstructure of the ceramics. Impedance spectroscopy measurements were performed in a wide frequency (1 Hz–40 MHz) and temperature (120–410 K) ranges under the flow of nitrogen.

## 2. Results and Discussion

**Figure 1:**
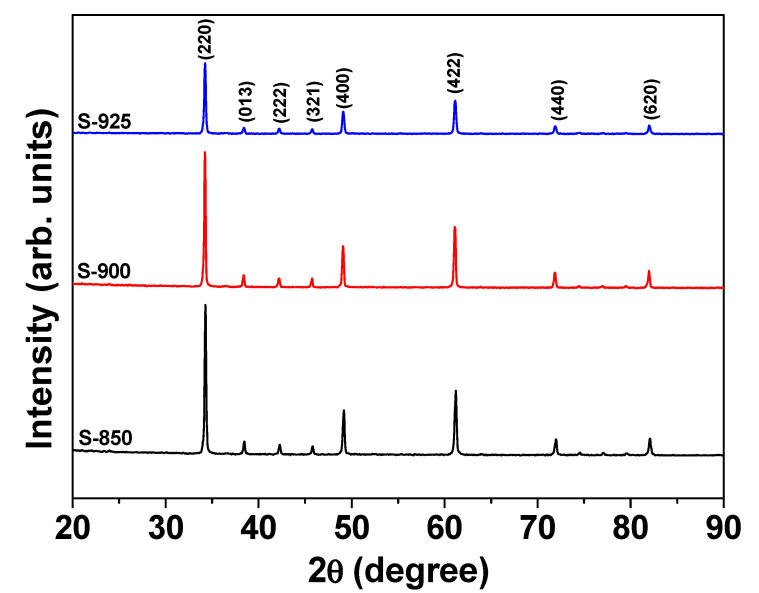
XRD patterns of the SPS NLCTO ceramics.

Room-temperature XRD patterns of the crushed pellets of NLCTO ceramics are shown in Figure 1. All the observed diffraction peaks are consistent with the cubic structure according to JCPDS#75-2188, with no secondary phase peaks. As can be seen in Figure 1, the diffraction peak (220) shifts toward a lower value of 2θ with increasing sintering temperature. This shift indicates an increase in the lattice constant of the ceramics with increasing sintering temperature. The lattice constants of the cubic phase for the investigated ceramics were calculated from the XRD patterns using the UnitCell program. The calculated values of the lattice parameter were found to be 7.413(5), 7.423(1), and 7.419(7) Å for the ceramics S-850, S-900, and S-925, respectively. These values are comparable to those reported for the NLCTO system [23,24]. During the SPS process, some Ti^4+^ ions convert into Ti^3+^ ions by capturing electrons resulting from oxygen vacancies [25]. Due to the difference in the ionic radii of Ti^4+^ and Ti^3+^, which are 0.605 and 0.670 Å [26], respectively, the lattice constant slightly increased with increasing sintering temperature. The densities of the current ceramics as measured from the mass and geometrical dimensions of the pellet were 5.03, 5.15, and 5.09 g/cm^3^ for S-850, S-900, and S-925, respectively. Besides, the theoretical density (ρ_theor_) was calculated using ρ_theor_= ZM/N_A_V, where Z is the number of atoms per unit cell (for body-centered cubic structures, Z = 2), M is the molecular weight corresponding to the chemical formula, N_A_ = 6.022 × 10^23^ mol^−1^ is Avogadro’s number, and V is the unit cell volume determined from X-ray measurements [27,28]. The calculated ρ_theor_ values were found to be 5.33, 5.32, and 5.33 for S-850, S-900, and S-925, respectively. Therefore, the experimental density of all the current samples was higher than 94% of the theoretical value.

**Figure 2:**
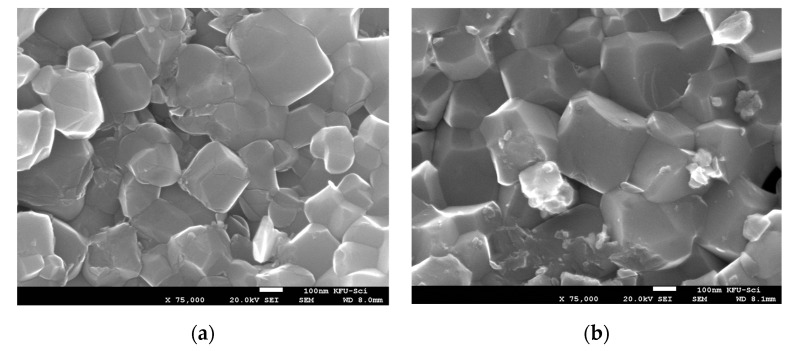

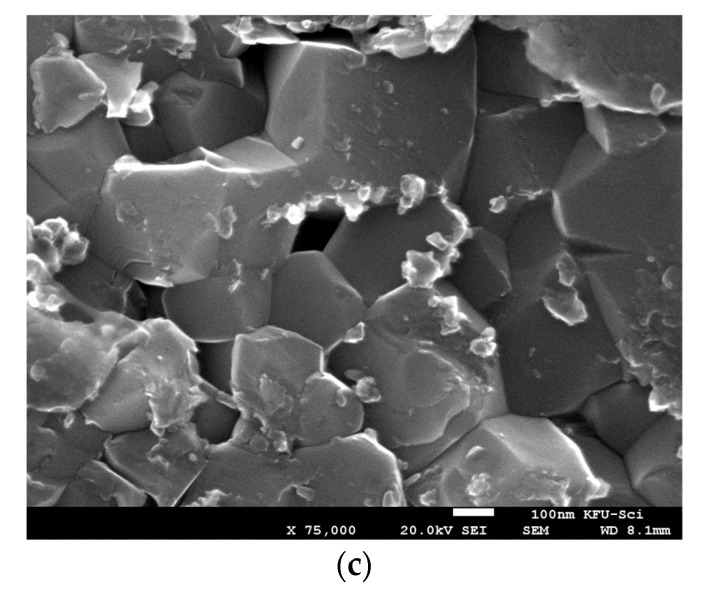
FE-SEM images of the scratched surface of SPS NLCTO ceramics: (**a**) S-850; (**b**) S-900; (**c**) S-925.

**Figure 3:**
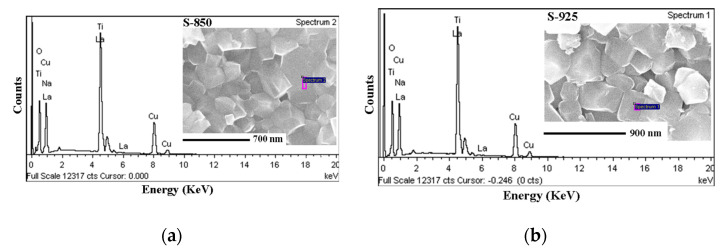
EDS analysis of the grain region of the ceramic samples: (**a**) S-850; (**b**) S-925.

FE-SEM images of the scratched surface of the sintered pellets are shown in Figure 2. The average grain size is 175 ± 30, 275 ± 25, and 300 ± 55 nm for the ceramics S-850, S-900, and S-925, respectively. These values are considerably smaller than the reported values for NLCTO ceramics prepared by SSR (~2–4 μm) [23] and modified SSR methods (~2–30 μm) [21]. The comparatively smaller grain size for SPS NLCTO ceramics is due to the fast heating rate and the short dwell time of the SPS process. Figure 3 shows the EDS spectra of the ceramic samples S-850 and S-975 as representative examples. Na, La, Cu, Ti, and O could be detected and were found to be homogeneously distributed in the prepared ceramics. The results of the elemental composition analysis are summarized in Table 1. Considering the experimental error (±2% to ±5%) in EDS measurements, it can be concluded that the chemical composition of the prepared samples is close to the theoretical formula.

**Figure 4:**
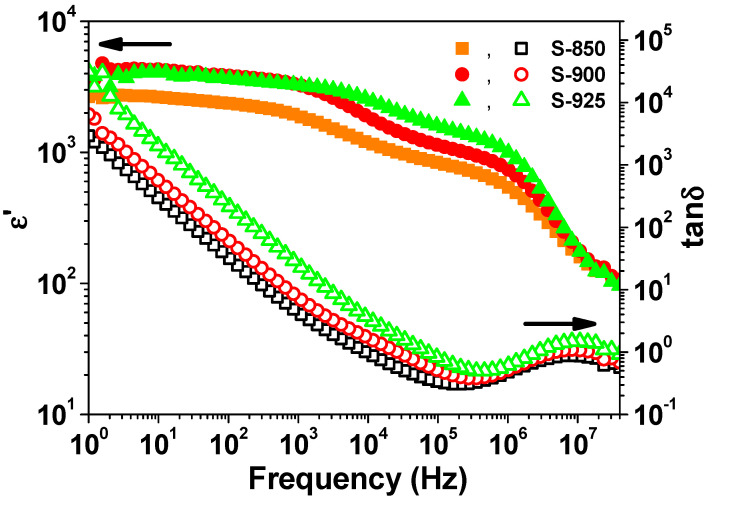
Room-temperature frequency dependence of ε′ and tan δ.

**Figure 5:**
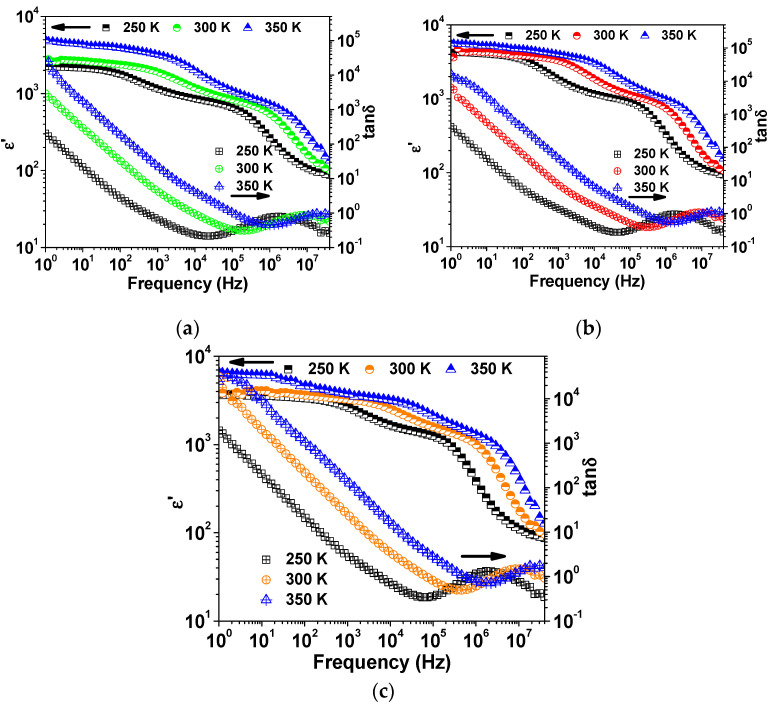
Frequency dependence of ε′ and tan δ at selected temperatures for (**a**) S-850, (**b**) S-900, and (**c**) S-925.

The frequency dependence of the dielectric constant (ε′) and dielectric loss (tan δ) at room temperature is depicted in Figure 4. All the samples showed colossal dielectric constants (ε′ > 10^3^) over most of the frequency range. As seen in Figure 4, with increasing sintering temperature, both ε′ and tan δ increase. Moreover, the sample S-900 showed the best compromise between the minimum tan δ (~0.39) and colossal ε′ (~1420) at 200 kHz. The dielectric constant of SPS NLCTO ceramics is comparable with the literature values [22,23]. Meanwhile, their dielectric losses are noticeably high compared to literature values. Figure 5 shows the frequency dependence of ε′ at selected measuring temperatures. The spectrum of ε′ at a given temperature of each sample was found to decrease with increasing frequency, accompanied by a relaxation peak in the spectrum of tan δ. It is seen that the relaxation peak shifts toward a higher frequency with increasing measuring temperature, which signifies a thermally activated process.

**Figure 6:**
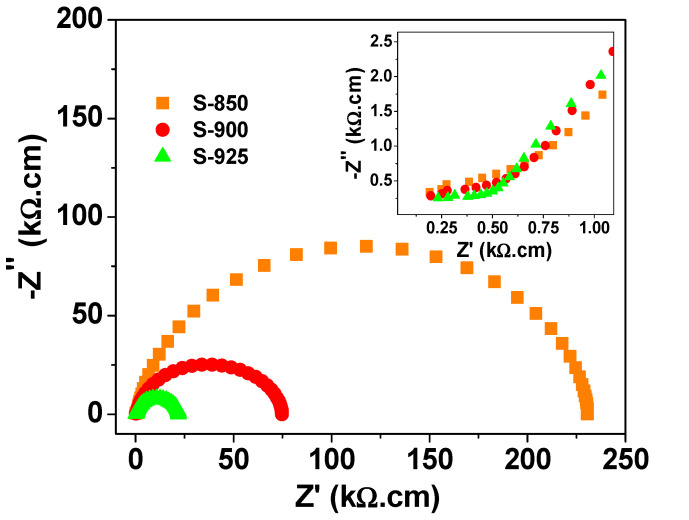
Nyquist plot of complex impedance for the SPS NLCTO ceramics. The inset represents a close-up of the high-frequency region of the plot.

**Figure 7:**
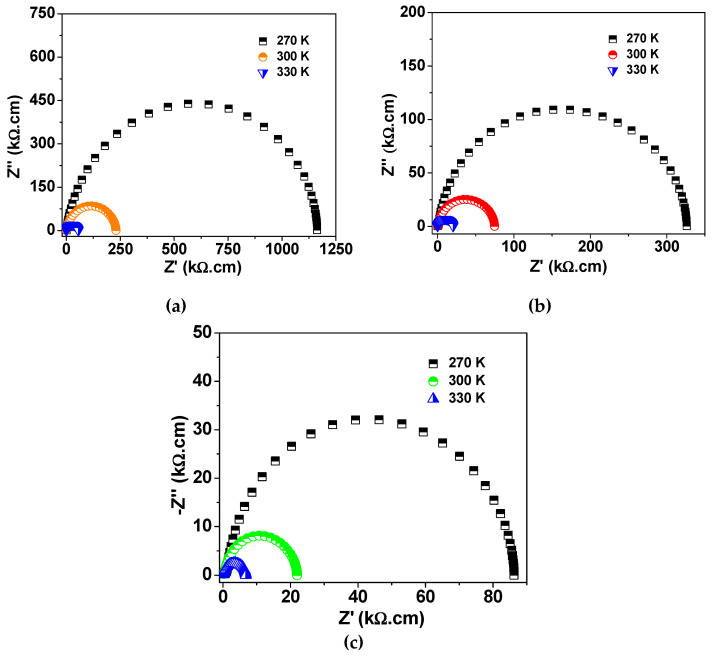
Nyquist plot of complex impedance at selected measuring temperatures for (**a**) S-850, (**b**) S-900, and (**c**) S-925.

Figure 6 shows the Nyquist plots of complex impedance at room temperature for the investigated NLCTO samples. The Nyquist plot of each sample is composed of two semicircles, which indicate the existence of two electrically active elements with considerable difference in resistivity. This structure is well presented by the internal barrier layer capacitance (IBLC) model [4,29]. According to the IBLC model, the high-frequency semicircle (inset of Figure 6) signifies the response of the grain while the larger semicircle is related to the response of the grain boundary. The resistivity values of the grain (R_g_) and the grain boundary (R_g.b._) could be obtained from the intercept of the relevant semicircle with the Z′-axis. The room temperature values of R_g_ and R_g.b._ are included in Table 2. R_g.b._ is about 2 orders of magnitude greater than R_g_ at room temperature for the current samples. Under the effect of an applied alternating voltage, charges are displaced from the less resistive grains toward the resistive grain boundary, where they pile up, thus forming the Maxwell–Wagner (M–W) interfacial polarization effect. This effect is thought to be responsible for the colossal dielectric constant of the current samples at low frequency. At high frequencies, charge carriers are not able to follow the variation in the applied alternating electric field. As a result, ε′ decreases due to the reduced polarizability. Moreover, the values of resistivity of the grain boundary for SPS NLCTO ceramics is less than the reported values for NLCTO ceramics prepared by other techniques [22,23]. This result explains the comparatively higher dielectric loss of the SPS NLCTO. The higher conductivity of SPS NLCTO ceramics is thought to be due to the reducing effect of vacuum during the SPS process. Besides, as shown in Figure 7, the resistivity of the samples decreases with increasing temperature, which indicates a semiconductor behavior.

**Figure 8:**
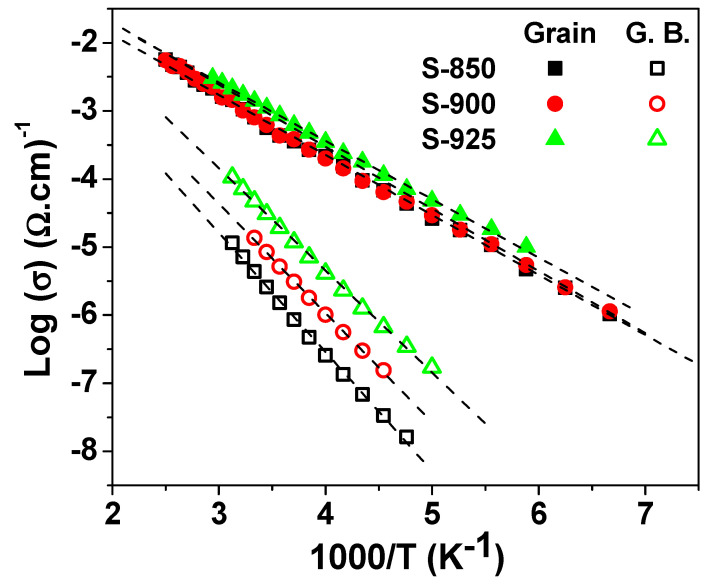
The Arrhenius plot for the conductivity of the grain and the grain boundary for the SPS NLCTO ceramics.

Figure 8 depicts the Arrhenius plot for the conductivity of the grain and the grain boundary of the current ceramics. Therefore, the electrical conduction mechanism is via the nearest-neighbor hopping according to the Arrhenius law of resistance, Equation (1):

(1) $$\sigma =\sigma_{0}\exp\left(\frac{-\Delta E}{k_{B}T}\right)$$

where σ_0_ is the pre-exponential factor, *k*_B_ is the Boltzmann constant, and ΔE is the activation energy for conduction. The obtained values for ΔE are summarized in Table 2. These values are on the order of 0.15 and 0.3 eV for the grain and the grain boundary, respectively.

**Figure 9:**
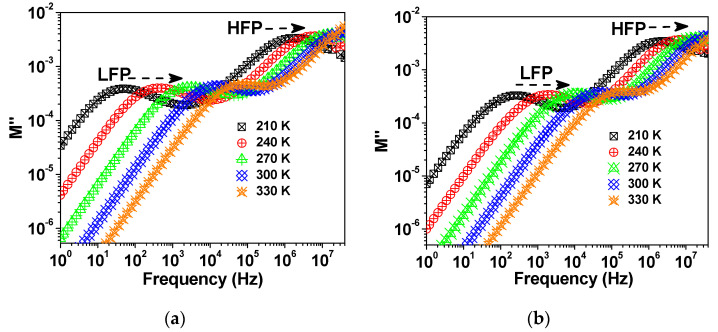

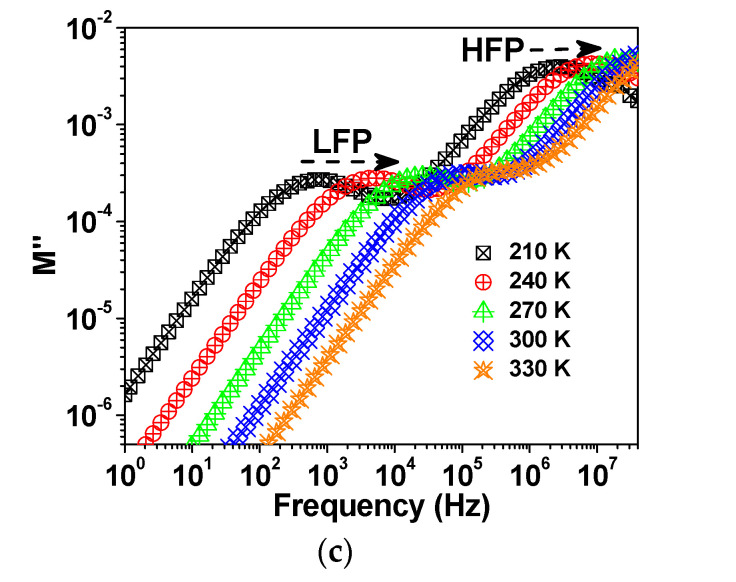
M″ spectra at selected temperatures for the samples (**a**) S-850, (**b**) S-900, and (**c**) S-925.

Figure 9 shows the spectra of M″ at selected measuring temperatures for each sample, where M″ is the imaginary part of the electric modulus M″ (M* = M′ + jM″ = 1/ε*, where ε* is the complex dielectric constant. Two relaxation peaks exist in the spectra of M″ at a given temperature. The height of the M″ peak is known to be inversely proportional to the capacitance C (M″/ε_0_ = 1/2C) [30], where ε_0_ is the permittivity of free space (ε_0_ = 8.854 × 10^−14^ F/cm). Therefore, the low-frequency peak (LFP) and the high-frequency peak (HFP) were attributed to the influence of the grain and the grain boundary, respectively, on conductivity relaxation of the samples. Both LFP and HFP peaks shifted toward high frequency with increasing temperature, which indicates that the relaxation time decreases with increasing temperature.

**Figure 10:**
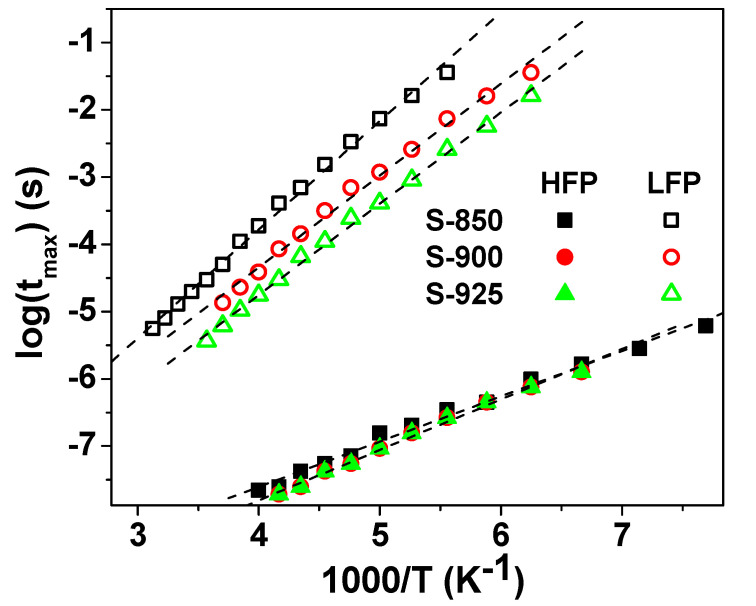
The Arrhenius plot for the relaxation time for the SPS NLCTO ceramics.

The mean value of the relaxation time, τ, was calculated from the peak frequency (f_max_) as τ = 1/2πf_max_. As seen in Figure 10, the temperature dependence of τ was found to fit the Arrhenius relation, Equation (2) [13,31]:

(2) $$\tau =\tau_{0}\exp\left(\frac{\Delta E_{R}}{k_{B}T}\right)$$

where τ_0_ is the pre-exponential factor, ΔE_R_ is the activation energy for the relaxation process, and k_B_ is the Boltzmann constant. The obtained values of ΔE_R_ are indicated in Table 2.

It can be seen that the activation energy values extracted from the complex plan plots and those extracted from the M″ spectra are close to each other. It is widely accepted that oxygen vacancies develop in titanium-based ceramics during high-temperature sintering due to the loss of oxygen [25]. Subsequently, both singly and doubly ionized oxygen vacancies would form. This process can be described simply by Equations (3) and (4), where the Kroger–Vink notation of defects is used.

(3) $$O_{o}\Leftrightarrow \frac{1}{2}O_{2}+V_{o}^{••}+2e^{-}$$

(4) $$V_{o}^{••}+e^{-}\Leftrightarrow V_{o}^{•}$$

where $V_{o}^{•}$ and $V_{o}^{••}$ are the singly and doubly ionized oxygen vacancies, respectively. The released electrons in this process may be captured by Ti^4+^ and/or Cu^2+^, thus forming of Ti^3+^ and Cu^+^ ions. It has been reported that the activation energy for the hopping of electrons among titanium ions of different valences (Ti^4+^/Ti^3+^) is ~0.13 eV [26]. Moreover, the activation energy for conduction and for relaxation caused by singly ionized oxygen vacancies has been frequently reported as 0.3–0.5 eV [32,33]. Considering the activation energies of the current study (Table 2), we believe that the LFP and HFP relaxation peaks in SPS NLCTO could be attributed to the electron hopping among multivalent ions and singly ionized oxygen vacancies, respectively.

## 3. Materials and Methods

Na_1/2_La_1/2_Cu_3_Ti_4_O_12_ (NLCTO) powder was synthesized by mechanochemical milling of stoichiometric amounts of high-purity La_2_O_3_, TiO_2_, CuO, and Na_2_CO_3_. The Fritsch P-7 premium line machine was used for 30 h at a rotation speed of 500 rpm using a 45 mL pot and balls made of tungsten carbide. Spark plasma sintering (SPS) was carried out under vacuum using the SPS 4–10 system (Thermal Technology LLC, Santa Rosa, CA, USA). The powder was confined in a 12 mm graphite die-punch system under 60 MPa pressure. Dense ceramics were obtained by sintering at 850 °C (S-850), 900 °C (S-900), and 925 °C (S-925) for 10 min. A heating rate of 200 °C/min was used for all the samples. The experimental density of the current ceramics was estimated from the mass and volume of the pellet. The phase composition was investigated by using a Stoe Stadi-P Image Plate, IP, (Stoe and Cie GmbH, Darmstadt, Germany), and Cu Kα1 radiation (λ = 1.5406 Å). The morphology and the elemental composition analysis were determined by using a field-emission scanning electron microscope (FE-SEM) (Joel, SM7600F, Tokyo, Japan) and an attached energy-dispersion X-ray spectroscope (EDS) system (Inca Oxford, High Wycombe, UK). The average grain size (D) of NLCTO ceramics was measured by the linear intercept method, given by D = 1.56 L/MN, where L is the random line length on the micrograph, M is the magnification of the micrograph, and N is the number of the grain boundaries intercepted by lines [34,35]. Impedance spectroscopy studies were performed on silver-paste-coated pellets in a wide frequency range (1–10 MHz) and at various temperatures (120–410 K) in a dry nitrogen atmosphere. A turnkey concept 50 system from Novocontrol was used for impedance spectroscopy (IS). The sample temperature was automatically controlled by Quatro Cryosystem.

## 4. Conclusions

Na_1/2_La_1/2_Cu_3_Ti_4_O_12_ ceramics were prepared by mechanochemical ball mill synthesis of the powder followed by SPS at 850–1025 °C for 10 min under vacuum. Structural characterization by XRD and SEM revealed a cubic phase with fine grain size (175–300 nm). SPS NLCTO ceramics showed colossal dielectric constants (ε′ > 10^3^) over most of the studied frequency range (1 Hz–40 MHz), but with comparatively high dielectric losses (tan δ > 0.1). Using Nyquist plots of the complex impedance and the modulus spectroscopy formalism, the SPS NLCTO ceramics were found to be electrically inhomogeneous and have two relaxation peaks. The relaxation peaks were attributed to the response of the grain and the grain boundary. The colossal dielectric constant and the high dielectric loss of the SPS NLCTO ceramics could be attributed to the combined effect of the internal barrier layer capacitance (IBLC) and the comparatively low resistivity of the grains.

## Figures and Tables

**Table 1:** Atomic % of the elements in the grain for NLCTO ceramics.

	Na	La	Ti	Cu	O
S-850	1.51	2.05	17.66	12.72	66.07
S-900	1.58	1.88	16.65	12.16	67.73
S-925	1.59	1.77	15.44	11.35	67.73

**Table 2:** The room temperature resistivity of the grain (R_g_) and the grain boundary (R_g.b._) and the activation energy for conduction (ΔE) and relaxation (ΔE_R_).

	R_g_ (kΩ.cm)	R_g.b._ (kΩ.cm)	ΔE (eV)		ΔE_R_ (eV)	
			Grain	G.B.	HFP	LFP
S-850	0.75	229	0.155	0.337	0.143	0.319
S-900	0.61	75	0.153	0.318	0.145	0.271
S-925	0.49	23	0.157	0.297	0.141	0.269

## Data Availability

The data presented in this study are available in the article.

## Sample Availability

Samples of the compounds are available from the authors on valid request.
